# Concurrent infection with porcine reproductive and respiratory syndrome virus and *Haemophilus parasuis* in two types of porcine macrophages: apoptosis, production of ROS and formation of multinucleated giant cells

**DOI:** 10.1186/s13567-017-0433-6

**Published:** 2017-05-04

**Authors:** Lenka Kavanová, Katarína Matiašková, Lenka Levá, Hana Štěpánová, Kateřina Nedbalcová, Ján Matiašovic, Martin Faldyna, Jiří Salát

**Affiliations:** 1 Veterinary Research Institute, Hudcova 296/70, 62100 Brno, Czech Republic; 2 Institute of Experimental Biology, Faculty of Science, Masaryk University, Kotlářská 267/2, 61137 Brno, Czech Republic; 3 University of Veterinary and Pharmaceutical Sciences Brno, Palackého třída 1946/1, 612 42 Brno, Czech Republic

## Abstract

Porcine reproductive and respiratory syndrome (PRRS) is one of the most significant and economically important infectious diseases affecting swine worldwide and can predispose pigs to secondary bacterial infections caused by, e.g. *Haemophilus parasuis*. The aim of the presented study was to compare susceptibility of two different types of macrophages which could be in contact with both pathogens during infection with PRRS virus (PRRSV) and in co-infection with *H. parasuis*. Alveolar macrophages (PAMs) as resident cells provide one of the first lines of defence against microbes invading lung tissue. On the other hand, monocyte derived macrophages (MDMs) represent inflammatory cells accumulating at the site of inflammation. While PAMs were relatively resistant to cytopathogenic effect caused by PRRSV, MDMs were much more sensitive to PRRSV infection. MDMs infected with PRRSV increased expression of pro-apoptotic Bad, Bax and p53 mRNA. Increased mortality of MDMs may be also related to a higher intensity of ROS production after infection with PRRSV. In addition, MDMs (but not PAMs) infected with *H. parasuis* alone formed multinucleated giant cells (MGC); these cells were not observed in MDMs infected with both pathogens. Higher sensitivity of MDMs to PRRSV infection, which is associated with limited MDMs survival and restriction of MGC formation, could contribute to the development of multifactorial respiratory disease of swine.

**Electronic supplementary material:**

The online version of this article (doi:10.1186/s13567-017-0433-6) contains supplementary material, which is available to authorized users.

## Introduction

Porcine reproductive and respiratory syndrome (PRRS) is one of the most significant and economically important infectious diseases affecting swine worldwide [1]. The causative agent is porcine reproductive and respiratory syndrome virus (PRRSV). It is an enveloped, positive-stranded RNA virus, belonging to the family *Arteriviridae*. PRRSV is associated with respiratory distress and reproductive failure in swine and poor growth performance in piglets [2].

The PRRSV targets cells of the porcine monocyte/macrophage lineage [3] where CD163 is the essential receptor for the virus infection [4, 5]. PRRSV primarily replicates in differentiated porcine alveolar macrophages (PAMs) [6, 7] but it has been identified in macrophages located in tissues, including lymph nodes, thymus, spleen, Peyer’s patches and liver [7, 8]. The expression of CD163 is dependent on the differentiation levels of monocyte lineage cells. Freshly isolated peripheral blood monocytes express extremely low levels of CD163 and are not susceptible to PRRSV infection [5] but as monocytes differentiate/age, the expression of CD163 increases and their susceptibility to PRRSV infection is enhanced [9]. Monocyte-derived macrophages (MDMs) [10] can be used as an alternative model for in vitro infection with PRRSV [5, 11, 12].

There are a number of published studies demonstrating that PRRSV induces apoptosis [13–15]. This highly regulated process is modulated by both pro-apoptotic and anti-apoptotic cellular factors. Two distinct pathways of apoptosis have been described: intrinsic and extrinsic apoptosis. Intrinsic apoptosis is initiated as a response to cellular stressors. The protein p53 is activated following DNA damage and triggers apoptosis through transcriptional activation of the Bcl-2 associated (Bax) gene [16]. It is still unclear whether PRRSV can induce apoptosis directly (within infected cells) or indirectly (within bystander cells) [16]. Cell apoptosis was mainly observed in PRRSV-inoculated MARC-145 cells [13, 14, 17, 18] and PAMs [19] but there is little information about mortality and cell apoptosis of PRRSV infected MDMs.

The PRRSV is considered to be one of the key etiological agents in multifactorial respiratory disease of swine. The virus can predispose pigs to infection by bacteria such as *Streptococcus suis*, *Haemophilus parasuis*, *Mycoplasma hyopneumoniae*, *Actinobacillus pleuropneumoniae* and *Salmonella* spp. [20–24]. The additive effect of PRRSV infection and a secondary bacterial infection in the induction of multifactorial respiratory diseases was described in the case of *H. parasuis* [25, 26].

Macrophages play an important role in the first line of defence against invading pathogens where production of reactive oxygen species (ROS) is one of the most important antimicrobial mechanisms. Oxidative stress caused by ROS has been suggested as an apoptosis mediator in virus-infected cells [27]. Increased ROS production was detected in the lungs of PRRSV-challenged pigs [28]. On the other hand, bacteria such as *Haemophilus influenzae* have evolved the OxyR system which coordinates the expression of numerous defensive antioxidants [29, 30].

Information about sensitivity of various types of macrophages to infection with PRRS virus in co-infection with *H. parasuis* is lacking. Viability of cells, virus replication, ROS production and apoptosis of different types of co-infected macrophages in vitro was analysed in this study in order to gain understanding of macrophage interactions with PRRSV and *H. parasuis* in multifactorial respiratory swine disease.

## Materials and methods

### Virus

The Lelystad strain of PRRSV (CAPM V-490) was obtained from the Collection of Animal Pathogenic Microorganisms (CAPM) at the Veterinary Research Institute (Brno, Czech Republic). The virus was propagated on the MARC-145 cell line and maintained in Dulbecco Modified Eagle’s Medium (DMEM) (Invitrogen) supplemented with 10% foetal bovine serum (FBS) (Thermo Scientific), 1% antibiotics (Antibiotic Antimycotic Solution 100×: 10 000 units penicillin, 10 mg streptomycin, and 25 μg amphotericin B per mL; Sigma-Aldrich) at 37 °C and 5% CO_2_. The virus was clarified by centrifugation, and its concentration was determined by plaque assay. The concentration of stock virus used in experiments was 5 × 10^6^ plaque forming units per mL.

### Bacteria

*Haemophilus parasuis* serotype 5, strain HP 132 (CAPM 6475) originating from a pig with meningitis was obtained from CAPM. Bacteria were grown on Mueller–Hinton agar broth with yeast extract and 5% sheep blood (LabMediaServis) overnight at 37 °C, and non-confluent growth was harvested and resuspended in calcium–magnesium free Dulbecco’s phosphate-buffered saline (D-PBS, Lonza). Bacteria were washed twice with D-PBS and resuspended in D-PBS, with the final concentration adjusted to optical density of 2.5 equivalent to 10^9^ CFU/mL, using a turbidimeter (DEN-1 McFarland densitometer, Biosan).

### Preparation of MDMs

CD14+ porcine monocytes were isolated from whole blood as described previously [31]. Peripheral blood mononuclear cells (PBMCs) were isolated from heparinized blood by Histopaque-1077 (Sigma-Aldrich) gradient. Monocytes were further enriched to a purity of >95% by positive magnetic bead selection (QuadroMACS™ cell separator, Miltenyi Biotec) using monoclonal antibody directed against CD14 (clone MIL2, AbD Serotec, Oxford, UK, 10 μL per 10^8^ cells) and goat anti-mouse IgG microbeads together with LS separation columns (Miltenyi Biotec). The obtained cells were cultured in 24-well plates at a concentration of 5 × 10^5^ cells per well in 1 mL of complete medium (DMEM with 10% FBS and 1% antibiotics) and incubated for 4 days at 37 °C in 5% CO_2_ to differentiate into macrophages. The cells for chemiluminescence assay were cultured in Nunc-Immuno™ MicroWell™ 96-well polystyrene plates (Sigma-Aldrich) at a concentration of 1 × 10^5^ in 250 µL of complete medium and incubated for 4 days at 37 °C in 5% CO_2_ to differentiate into macrophages.

### Preparation of PAMs

PAMs were collected as described previously [32] by bronchoalveolar lavage from five 6 to 8 week-old pigs from a PRRSV negative herd. The use of animals was approved by the Ethical committee of Ministry of Agriculture (approval protocol No. MZe 1487) as a part of project Respig (QJ1210120). Briefly, pigs were euthanized with the intravenous injection of the anaesthetic T61 (Intervet International B.V.) based on body weight according to the manufacturer’s recommendations (5 mL/50 kg of body weight) and necropsied. The trachea and lungs were immediately removed, and the lungs were flushed with D-PBS. The aliquots with PAMs were frozen in a medium containing 75% RPMI-1640, 20% FBS and 5% dimethylsulphoxide (DMSO) (Sigma-Aldrich) and stored in liquid nitrogen until use. PAMs were thawed in a water bath at 37 °C before each experiment. Cell viability after the freeze/thaw process as determined by trypan blue exclusion was higher than 90%. The cells were washed by DMEM prior to use in the experiments. Porcine alveolar macrophages were placed into 24-well polystyrene culture plates at a concentration of 5 × 10^5^ cells per well in 1 mL of complete medium (DMEM with 10% FBS and 1% antibiotics) and incubated overnight at 37 °C in 5% CO_2_.

### Flow cytometry

Differentiation of MDMs and differences in the expression of the surface molecule CD163 between MDMs and PAMs were evaluated by flow cytometry. The cells were harvested by 0.2% EDTA in PBS, washed in PBS and labelled with the following unlabelled primary antibodies against surface protein: anti-CD163 (2A10/11, IgG1, Bio-rad). AlexaFluor 488-conjugated mouse IgG1 or IgG2a isotype-specific goat antisera (Invitrogen) were used as the secondary antibodies. Control samples were stained with secondary antibody only. Flow cytometry was performed using a LSR Fortessa flow cytometer operated by Diva software (Becton–Dickinson). Data are shown as median fluorescence intensity ratio (MFI ratio); MFI ratio = MFI of specific Ab-stained cells + AlexaFluor 488-conjugated mouse IgG1 (CD163)/MFI of control cells stained only with AlexaFluor 488-conjugated mouse IgG1 or IgG2a isotype-specific secondary antibody. The experiment was performed using PAMs/MDMs isolated from four pigs.

### Experimental design and sampling

Prepared macrophages were washed with complete medium and infected with PRRSV in multiplicity of infection (MOI) 0.5 at 24 h after seeding (PAMs) or immediately after differentiation (MDMs). The medium from uninfected MARC-145 cells was used as mock-infection, and complete medium alone was used as a control. Twenty-four hours post-infection (PI) with PRRSV, macrophages were washed to remove antibiotics and subsequently infected with *H. parasuis* (MOI 10). Dulbecco’s PBS was used as a control solution in groups without *H. parasuis*. The following groups were included in the trial: (1) PRRSV + *H. parasuis* infected, (2) PRRSV infected, (3) mock + *H. parasuis* infected, (4) mock infected, (5) *H. parasuis* infected, and (6) non-infected control. The culture supernatants were collected at 4 h PI with *H. parasuis*/28 h PI with PRRSV (4/28 h PI) and 24 h PI with *H. parasuis*/48 h PI with PRRSV (24/48 h PI) for the evaluation cell mortality early (4/28 h) or late (24/48 h) after infection with the bacterium/virus. Total RNA was extracted from the harvested cells at the same experimental time points. Five independent experiments including culture duplicates were conducted, using PAMs/MDMs isolated from five pigs.

### Cell mortality

Mortality of infected cells was detected using the CytoTox 96 Non-Radioactive Cytotoxicity assay (Promega) following the manufacturer’s instructions.

### PRRSV and *H. parasuis* quantification

PRRSV replication in PAMs/MDMs was determined by virus titration method. The growth of bacteria was measured by spectrophotometry (A_600_ nm) according to Bello-Ortí et al. [33] and Lichtensteiger and Vimr [34]. Cells were infected with PRRSV at MOI 0.5 and incubated with the virus for 2 h at 37 °C in 5% CO_2_. The cells were washed once and the complete medium was added. Twenty-four hours PI with PRRSV were macrophages infected with *H. parasuis* (MOI 10). Cells were frozen at −80 °C at 0, 28, 48, 72, 96 h PI with PRRSV and 4, 24, 48 and 72 h PI with *H. parasuis* for the evaluation of PRRSV titres and bacterial growth. The supernatant was assayed using the standard method on MARC-145 cells. The virus titres were expressed as TCID_50_/mL (50% tissue culture infectious dose per mL) to examine the virus replication in PAMs or MDMs. TCID_50_/mL was calculated by the Spearman & Kärber algorithm as described in Hierholzer and Killington [35]. Cytopathic effect was observed using light microscopy. Microscopy was performed using a microscope Olympus IX51. Supernatant for evaluation of bacteria growth was centrifuged at 6000 *g* for 10 min, the pellets was resuspended in DPBS and absorbance at 600 nm was measured.

### Real-time RT-PCR for the detection of apoptosis related genes mRNA

Bad, Bax, p53 and Bcl-2 expression were quantified by real-time RT-PCR. RNA was isolated from harvested PAMs/MDMs using the RNeasy Mini Kit (Qiagen) following manufacturer’s instructions. M-MLV reverse transcriptase (Invitrogen) and oligo-dT primers (Generi Biotech) were used for reverse transcription. Measurements were performed using the QuantiTect SYBR Green PCR Kit (Qiagen) and gene specific primers (Generi Biotech) (Table 1) on a LightCycler 480 II with a 384-well plate block (Roche). Primers were designed using NCBI primer designing tool (http://www.ncbi.nlm.nih.gov/tools/primer-blast/). Hypoxanthine phosphoribosyltransferase (HPRT) was evaluated as the most constitutively expressed gene in our samples using RefFinder tool (http://www.leonxie.com/referencegene.php) and was selected to adjust mRNA measurements. The other tested genes which showed less stabile transcription were: TATA binding protein 1 and hydroxymethylbilane synthase. The threshold cycle values (Ct) of the genes of interest were first normalized to the Ct value of HPRT reference mRNA (ΔCt), and the normalized mRNA levels were calculated as 2(^−ΔCt^). The results are presented as mean values of fold increase of the gene of interest.

**Table 1:** Primers used for real time PCR quantification of gene expression.

	Sequence 5′–3′	Reference
Bad-For	CTG GGC TGC ACA GCG TTA T	This study
Bad-Rev	GGC GAG GAA GTC CCT TCT TG	This study
Bcl2-For	AGT ACC TGA ACC GGC ACC TG	This study
Bcl2-Rev	CAG CCA GGA GAA ATC AAA TAG AGG	This study
Bax-For	AAC ATG GAG CTG CAG AGG ATG	This study
Bax-Rev	GTT GCC GTC AGC AAA CAT TTC	This study
HPRT-For	GAG CTA CTG TAA TGA CCA GTC AAC G	[32]
HPRT-Rev	CCA GTG TCA ATT ATA TCT TCA ACA ATC AA	[32]
p53-For	AAA AGA AGA AGC CAC TGG ATG G	This study
p53-Rev	GTC ATT CAG CTC TCG GAA CAT CT	This study

### Fluorescence microscopy

The cells were grown on 24 well-plates as described above. After 4 h and 24 h PI with *H. parasuis*, the cells were fixed with 4% paraformaldehyde, macrophages were labelled with DAPI (nuclear stain) (Sigma-Aldrich) and with Alexa Fluor (AF) 594 conjugated phalloidin (Life Technologies) to visualize the actin cytoskeleton. Microscopy was performed using an epifluorescence inverted microscope Olympus IX51 equipped with a LUCPlanFLN 40 × (NA 0.60) objective using fluorescence mode to detect DAPI and AF-594.

### Chemiluminescence assay

Production of reactive oxygen species of experimentally infected PAMs and MDMs was measured using chemiluminescence (CL). The assay was performed in Nunc-Immuno™ MicroWell™ 96-well polystyrene plates (Sigma-Aldrich). Cells were seeded in DMEM with 1% antibiotics and 10% FBS at a concentration of 1 × 10^5^ cells per well. The cells were incubated overnight at 37 °C in 5% CO_2_. The medium with non-adherent cells was removed, and cells were infected with PRRSV at MOI 0.5 or the mock infection solution or the control medium was added. Cells were washed with Hanks’ balanced salt solution (HBSS, Lonza) 24 h and 48 h PI with PRRSV. Luminol-derivative L-012 (Wako Chemicals GmbH) was added to amplify the CL induced by ROS of stimulated cells. L-012 was diluted in HBSS to the final concentration of 0.15 mmol/L. A suspension of *H. parasuis* (MOI 10) was then added to the cell culture containing luminol L-012, and the plate was centrifuged at 250*g* for 5 min. The following groups were included: (1) PRRSV + *H. parasuis* infected, (2) PRRSV infected, (3) mock + *H. parasuis* infected, (4) mock infected, (5) *H. parasuis* infected, and (6) non-infected control. Chemiluminescence was measured immediately after adding *H. parasuis* at 37 °C using a multidetection microplate reader Synergy H1 (BioTek) in kinetic mode for 2 h. The results are expressed as integrals of chemiluminescence intensity (per 1 × 10^5^ viable cells) induced in PAMs/MDMs with infection(s), and data are presented as percentage relative to the non-infected control. The viability of cells was measured using the CCK-8 kit (Sigma-Aldrich), following manufacturer’s instructions. Five independent experiments including culture triplicates were performed using PAMs/MDMs isolated from five pigs.

### Statistical analysis

The normality of data distribution was confirmed by the Shapiro–Wilk’s W test, and homogeneity of variances by the Levene’s test. Experimental groups were compared with Student’s *t* test (mortality, multinucleated giant cells) or using a non-parametric test for paired samples, (Wilcoxon signed-rank test; flow cytometry, *H. parasuis* growth, expression of apoptosis related genes, chemiluminescence assay). A *p* value of < 0.05 was considered significant, unless otherwise stated. Data were analysed using Statistica 12 (StatSoft).

## Results

### Flow cytometry

**Figure 1:**
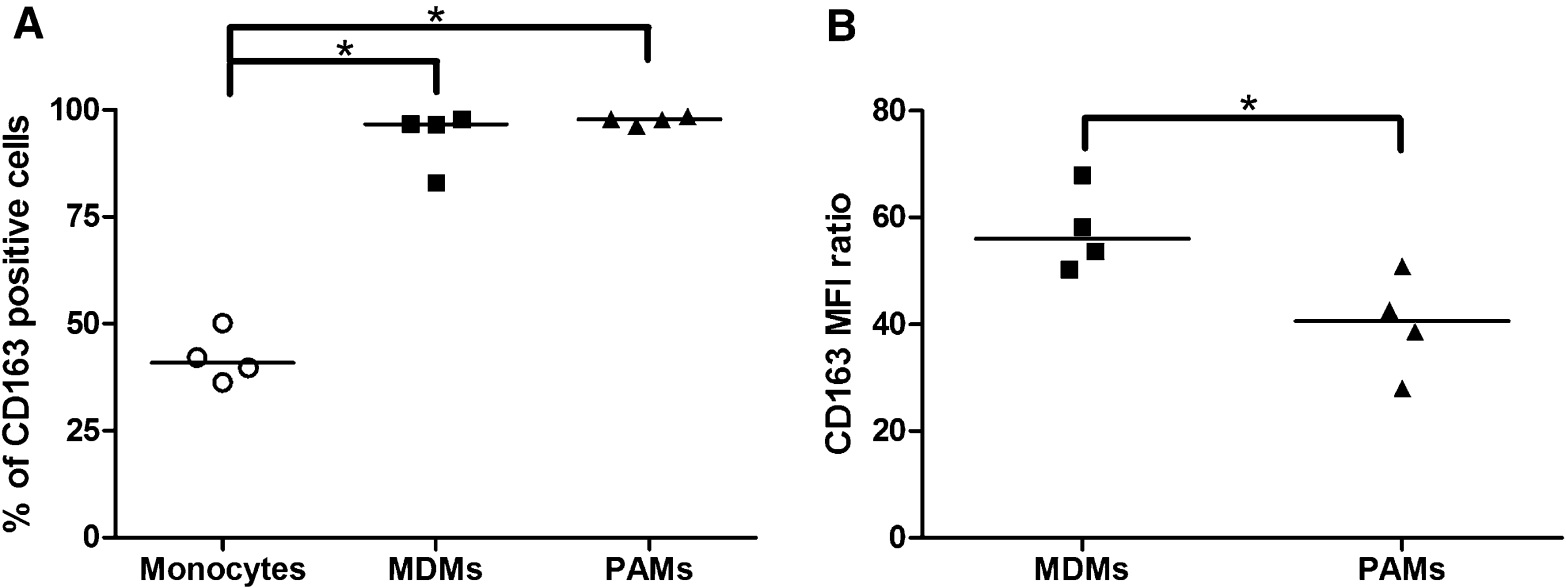
**Surface marker CD163 on monocytes, MDMs and PAMs.** Percentage of positive cells (**A**) and MFI ratio: the ratio of the MFI of the positive population in a sample to its corresponding isotype control (**B**) was measured by flow cytometry. Data are presented as median of four animals and each dot represents the result of an individual pig. Significant differences between tested groups are denoted by *(*p* < 0.05).

Peripheral blood monocytes differentiated within 4 days of culture. Differentiation was confirmed by elevated amounts of CD163(+) population from 42.1 ±5.9% to 93.6 ± 7.0% (*p* < 0.05) (Figure 1A) (Additional file 1).

The difference between the two macrophage types was further observed by the flow cytometry analysis (Figure 1). The percentage of CD163 positive cells in MDMs was similar to percentage of positive cells in PAMs (Figure 1A). On the other hand, the expression of CD163 on MDMs was significantly higher (*p* < 0.05) than on PAMs (Figure 1B).

### Mortality of infected MDMs/PAMs

**Figure 2:**
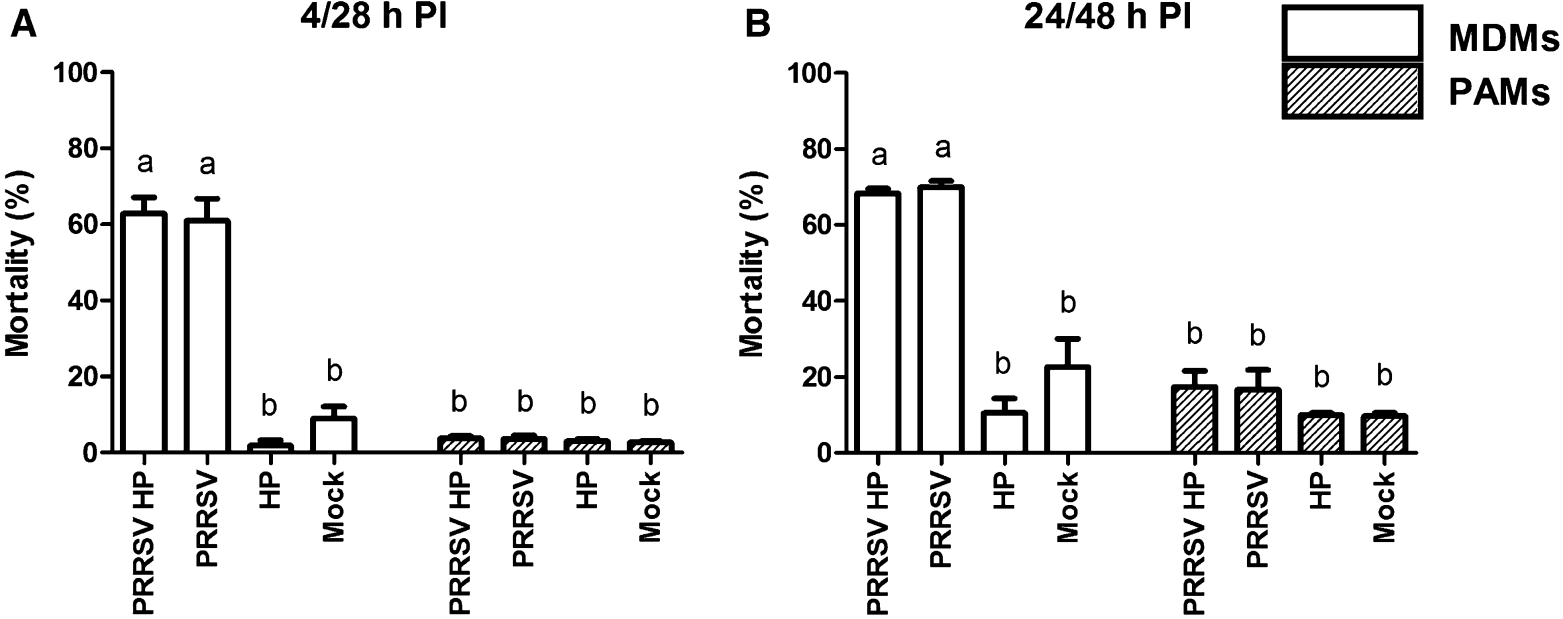
**Mortality of macrophages infected with PRRSV and** ***H. parasuis*** **(HP).** Mortality of MDM and PAMs was measured using CytoTox 96 non-radioactive Cytotoxicity assay at 4/28 h (**A**) and 24/48 h (**B**) PI with PRRSV and *H. parasuis*. Data are the mean ± SEM of three independent experiments. Significant differences between tested groups are denoted by different letters (a, b) (*p* < 0.05).

The average mortality rates of non-infected/mock infected MDMs were 9.1 ± 7.6% and 22.6 ± 18.2% at 4/28 and 24/48 h, respectively. Mortality of the PRRSV infected MDMs was significantly increased to 61.0 ± 14.3% and 70.0 ± 3.7% at 4/28 and 24/48 h PI. The mortality was similar in the *H. parasuis* infected group and in non-infected/mock infected groups (2.2 ± 3.9% and 7.7 ± 11.9% PI with *H. parasuis*) (Figure 2A). *H. parasuis* did not affect mortality of simultaneously infected groups in both types of cells (Figure 2).

Mortality of infected PAMs was published in previous study [26]. Briefly, the mortality of non-infected/mock infected PAMs was 2.7 ± 0.6% and 9.7 ± 1.5% at 4/28 and 24/48 h PI, respectively. Observed mortality of PRRSV infected PAMs was 3.7 ± 1.5% and 16.7 ± 9.3% at 4/28 and 24/48 h PI, respectively. *H. parasuis* infection was not associated with increased mortality of infected PAMs (3.0 ± 1.0% and 10.0 ± 1.7% at 4/28 and 24/48 h PI with *H. parasuis)* (Figure 2B).

All obtained results of the (3) mock-infected + *H. parasuis* group and the (4) mock infected group did not differ from (5) *H. parasuis* infected group and (6) non-infected control group, respectively. Therefore, only (1) PRRSV + *H. parasuis* infected, (2) PRRSV infected, (3) mock + *H. parasuis* infected, (4) and mock infected groups of PAMs/MDMs were included in the (Figure 2), likewise the results regarding the expression of apoptosis related genes and chemiluminescence assay.

### PRRSV replication in MDMs/PAMs

**Figure 3:**
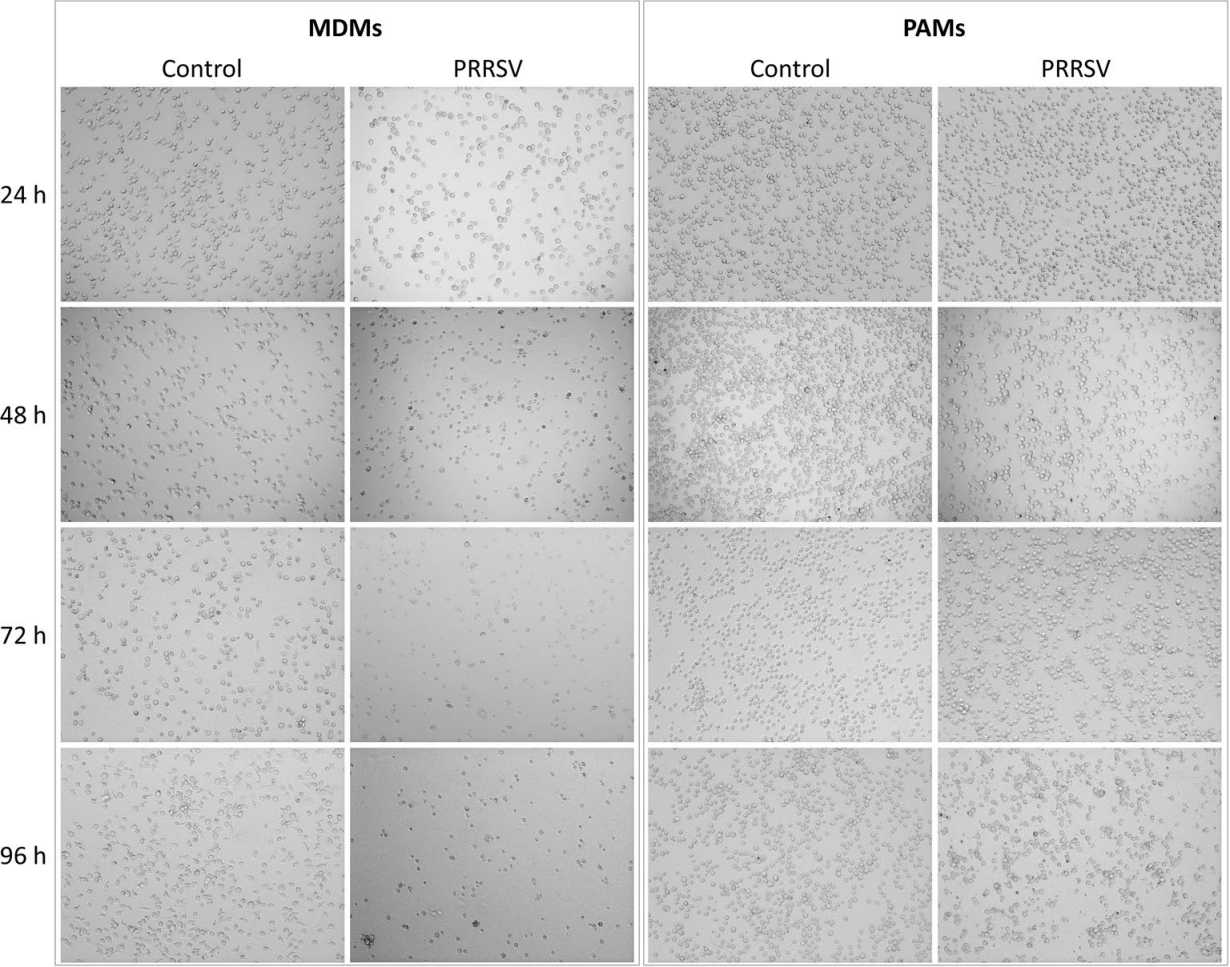
**Viability of MDMs and PAMs infected with PRRSV.** Cells were infected with PRRSV and observed by light microscopy at 24, 48, 72 and 96 h PI.

Cell culture medium was collected at 24, 48, 72 and 96 h to determine if elevated mortality of MDMs is related to virus replication. Our results show that PRRSV replicated in a similar pattern in both cell types (Table 2). The highest PRRSV titres were detected in culture supernatants of infected PAMs and MDMs at 24 h PI. Cell viability after PRRSV inoculation was observed by light microscopy. Decline of MDMs viability was observed after 24 h PI with PRRSV, while a decreased number of PAMs was observed after 96 h PI with PRRSV (Figure 3). Replication of virus was not affected by presence of *H. parasuis* (data not shown).

**Table 2:** Viral titres in the supernatant of PRRSV infected cultures from three independent experiments performed in culture triplicates are expressed as TCID_50_/mL.

	Time post infection with PRRSV							
	24 h		48 h		72 h		96 h	
MDMs	3.78 × 10^5^	+sd 2.00 × 10^5^	6.31 × 10^4^	+sd 3.15 × 10^4^	1.76 × 10^4^	+sd 7.66 × 10^3^	1.76 × 10^4^	+sd 1.08 × 10^4^
		−sd 1.31 × 10^5^		−sd 2.10 × 10^4^		−sd 5.33 × 10^3^		−sd 6.68 × 10^3^
PAMs	3.78 × 10^5^	+sd 2.32 × 10^5^	6.31 × 10^4^	+sd 3.88 × 10^4^	2.93 × 10^3^	+sd 1.18 × 10^3^	1.36 × 10^3^	+sd 7.19 × 10^2^
		−sd 1.44 × 10^5^		−sd 2.40 × 10^4^		−sd 8.41 × 10^2^		−sd 4.70 × 10^2^

### Growth of *H. parasuis*

**Figure 4:**
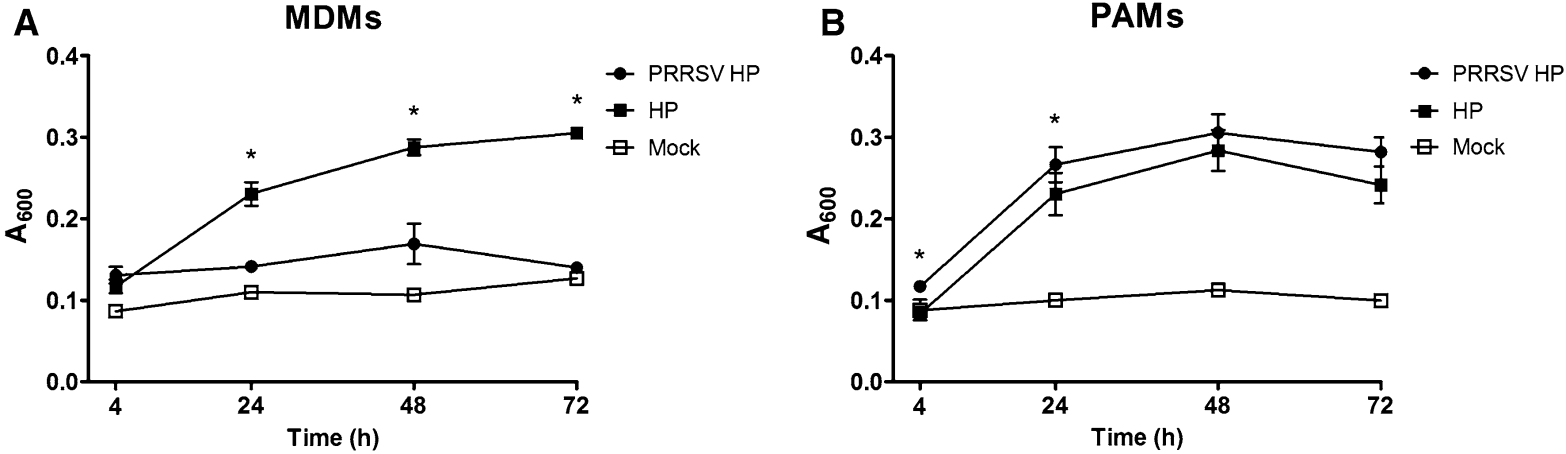
**Growth of** ***H. parasuis*** **on MDMs (A) or PAMs (B) was measured by spectrophotometry (A**_**600**_ **nm).** Mock curves serve as a baseline which includes cell debris from cultivated macrophages after freezing and thawing process. Data are presented as mean with SEM of three animals measured in culture duplicates. Significant differences between co-infected cells and cells infected with *H. parasuis* only are denoted by *(*p* < 0.05).

*Haemophilus parasuis* growth was determined by spectrophotometry. *H. parasuis* added to the complete medium without antibiotics had absorbance at the background level at all assay times (data not shown). Growth of *H. parasuis* in wells with MDMs was affected by the presence of virus (Figure 4A). In the wells with *H. parasuis* infected MDMs were detected higher amounts of bacteria compared to wells with co-infected MDMs. On the other hand, the amount of *H. parasuis* in PAMs infected with both pathogens was higher or on the same levels as in PAMs infected only with bacteria (Figure 4B).

### Expression of apoptosis related genes

The influence of co-infection with PRRSV and *H. parasuis* on expression of apoptosis related genes was characterized in two types of macrophages. The degree of target gene expression was calculated as fold-expression of the reference gene hypoxanthine phosphoribosyltransferase (HPRT). Apoptosis was analysed using relative quantification of typical pro-apoptotic gene expression markers including Bad, Bax, p53 and anti-apoptotic gene Bcl-2.

### MDMs

**Figure 5:**
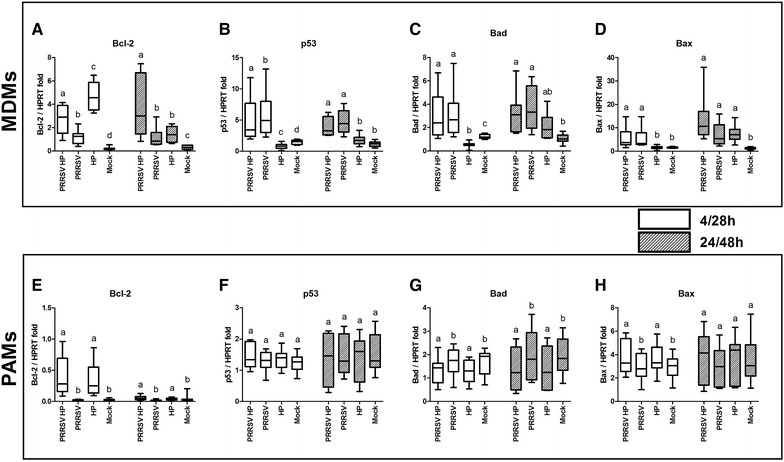
**Expression rates of mRNA of selected apoptosis-related genes.** Selected anti-apoptotic Bcl-2 (**A**, **E**) and pro-apoptotic p53 (**B**, **F**), Bad (**C**, **G**) and Bax (**D**, **H**) genes were measured by real-time PCR using gene specific primers at 4/28 and 24/48 h PI in MDMs (**A**, **B**, **C**, **D**) or PAMs (**E**, **F**, **G**, **H**). Boxplots indicate the median (middle line), 25th and 75th percentiles (boxes), and maximum and minimum (whiskers) of five independent experiments performed in culture duplicates. The degree of gene expression is displayed as fold change of the housekeeping gene hypoxanthine phosphoribosyltransferase (HPRT) expression. Mock-infected MDMs/PAMs served as controls for PRRSV infection. Significant differences between PRRSV and *H. parasuis* co-infected cells and other tested groups are denoted by different letters (a, b, c, d) (*p* < 0.05).

*Haemophilus parasuis* only and PRRSV only infected MDMs showed significant (*p* < 0.05) upregulation of the anti-apoptotic molecule Bcl-2 compared to controls at both assay times (Figure 5A). However, combined infection of MDMs with both pathogens or PRRSV only showed significantly (*p* < 0.05) lower expression of Bcl-2 mRNA in comparison with *H. parasuis* only infected MDMs at 4/28 h PI. On the other hand, simultaneously infected cells showed the highest expression (*p* < 0.05) of Bcl-2 mRNA at 24/48 h PI.

Although in both groups infected with PRRSV a significant increase of the pro-apoptotic molecule p53 mRNA was induced at both assay times (Figure 5B), the concurrent infection triggered a significantly lower response (*p* < 0.05) than in the PRRSV only infected group at 4/28 h PI. Expression of p53 mRNA was significantly down-regulated (*p* < 0.05) after infection with *H. parasuis* alone compared to the mock infected group at 4/28 h PI.

The observed expression pattern of Bad mRNA (Figure 5C) was similar to that of p53 at 4/28 h PI, with the significantly decreased (*p* < 0.05) production of mRNA in *H. parasuis* infected MDMs in comparison with mock treated cells. The highest production of Bad mRNA (*p* < 0.05) was detected in PRRSV infected groups at both assay times. *H. parasuis* infection did not change the level of Bad mRNA expression compared to mock infected cells at 24/48 h PI.

Both groups infected with PRRSV, but not with *H. parasuis* alone, showed increased (*p* < 0.05) production of Bax mRNA at 4/28 h PI (Figure 5D). The increased level of Bax mRNA was detected after infections with both pathogens compared to mock infection at 24/48 h PI.

Co-infection of MDMs with both pathogens did not influence the expression of Bad and Bax mRNA at both assay times compared to appropriate control.

### PAMs

*Haemophilus parasuis* infected PAMs induced significantly higher expression of anti-apoptotic Bcl-2 mRNA in comparison with mock infected PAMs at both assay times (Figure 5E). Expression of pro-apoptotic p53 mRNA was unchanged in all infected groups at both assay times (Figure 5F).

Decreased expression of pro-apoptotic Bad mRNA was observed at both assay times (Figure 5G). However, expression of the pro-apoptotic gene for Bax was up-regulated at 4 h PI with *H. parasuis* (Figure 5H).

The results showed that pro-apoptotic genes like Bad and Bax were not overexpressed after infection with PRRSV in PAMs (Figures 5G and H). PRRSV did not influence the level of Bcl-2 mRNA expression compared to mock infected cells (Figure 5E).

Combined infection with both pathogens did not change the levels of mRNA expression of apoptosis related genes compared to appropriate controls.

### Multinucleated giant cells

**Figure 6:**
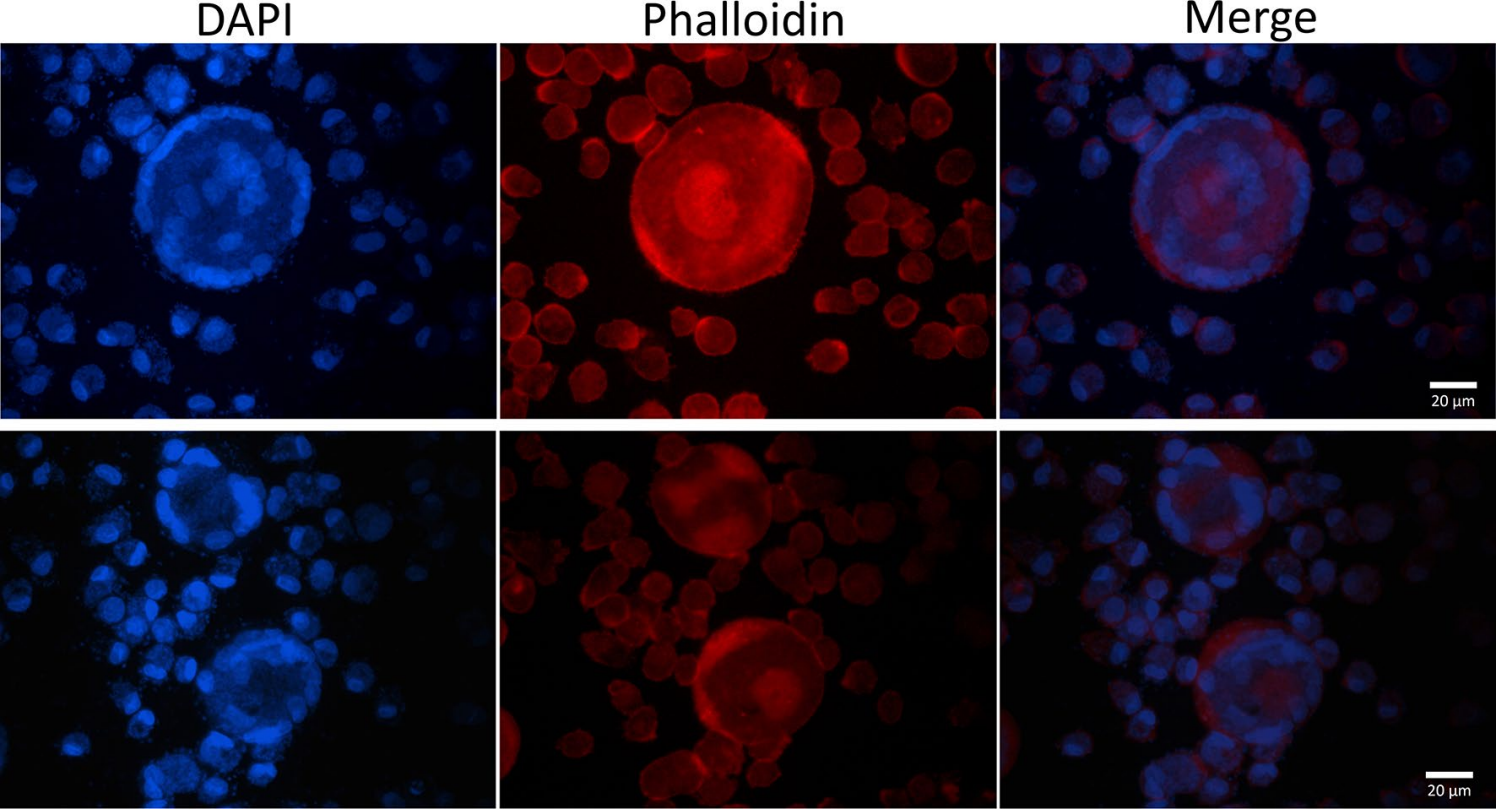
**Typical examples of multinucleated giant cells of MDMs infected with** ***H. parasuis*** **(24** **h PI).** MDMs were stained with DAPI to label the nuclei (blue) and with Alexa Fluor 594 conjugate phalloidin to label the cytoskeleton (red).

The cells were monitored throughout the experiment by light microscopy. Control MDMs and mock-infected cell did not change their morphology during the experiment. Excessively large cells were observed by light microscopy in the group of MDMs infected with *H. parasuis* at 24 h PI. Therefore, fluorescence microscopy was used and multinucleated giant cells (MGC) (Figure 6) were detected. The number of MGC induced by the bacterium was determined under a microscope (400 ×). Results showed that the number of nuclei per 100 cells was 102 ± 2.6 in non-infected and 160.2 ± 23.8 in *H. parasuis* infected MDMs (*p* < 0.05). The percentage of MGC induced by *H. parasuis* was 13.6 ± 2.8%. MDMs infected with PRRSV or simultaneously infected with PRRSV and *H. parasuis* did not form MGC. Formation of MGC in PAMs was not observed (101.5 ± 1.2 and 101.3 ± 0.6 nuclei per 100 cells in non-infected and *H. parasuis* infected cells, respectively). Data shown represents means and standard errors of three independent experiments.

### Chemiluminescence assay

**Figure 7:**
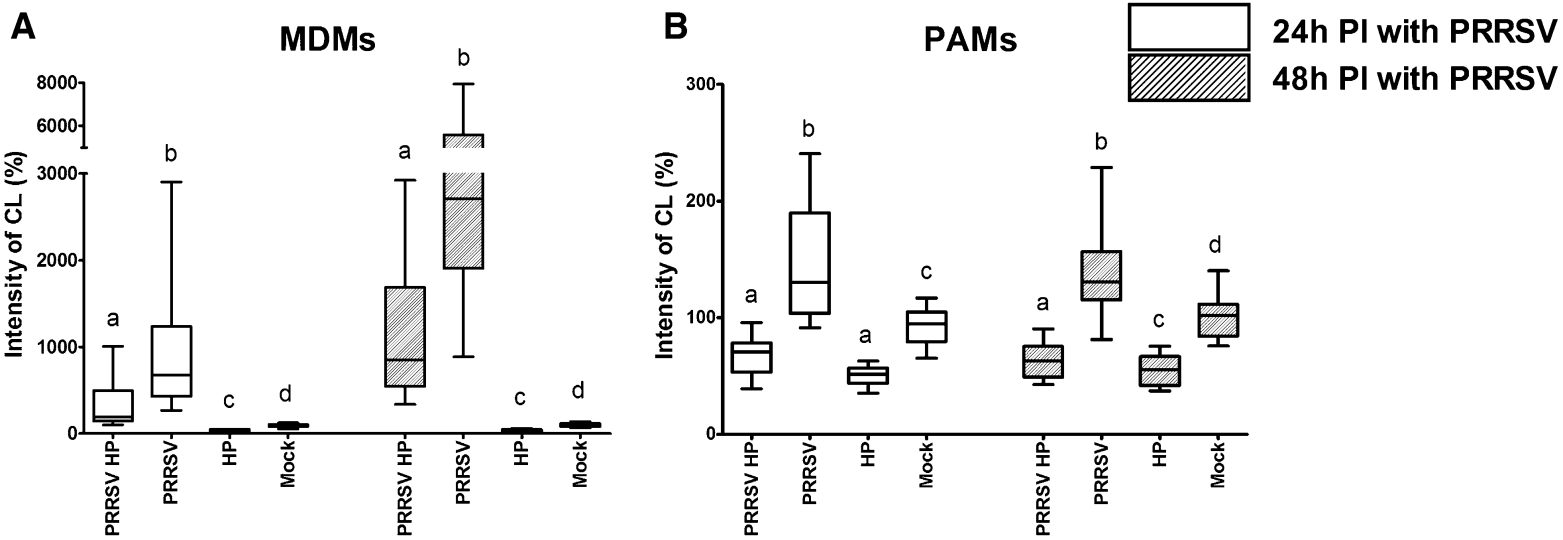
**ROS production. ROS produced by MDMs (A) or PAMs (B) were measured by chemiluminescence assay at 24 and 48** **h PI with PRRSV.** Infection with *H. parasuis* was performed immediately prior to measurement. Data are presented as the percentage of CL levels in non-infected control. Boxplots indicate the median (middle line), 25th and 75th percentiles (boxes), and maximum and minimum (whiskers) of five independent experiments performed in culture triplicates. Significant differences between tested groups are denoted by different letters (a, b, c, d) (*p* < 0.05).

Comparison of reactive oxygen species production by the co-infected, individually (PRRSV or *H. parasuis*) infected, and mock-infected macrophages is shown in Figure 7.

The highest production of ROS by MDMs was detected in the PRRSV infected group at 24 and 48 h PI (Figure 7A). On the other hand, *H. parasuis* reduced the production of ROS in comparison with relevant control groups at both assay times. MDMs infected with both pathogens showed decreased levels of released ROS in comparison with PRRSV infected groups at both assay times.

The observed ROS production in MDMs after infection with PRRSV and *H. parasuis* was similar to PAMs (Figure 7B). Significantly enhanced (*p* < 0.05) production of ROS was detected in the PRRSV infected group of PAMs at 24 and 48 h PI. Conversely, *H. parasuis* infection significantly reduced (*p* < 0.05) the intensity of the detected CL response compared to the mock-infected group. A significant (*p* < 0.05) reduction in ROS production was also observed in the co-infected group at 24 and 48 h PI compared with PRRSV-only and mock-infected groups.

The highest level of released ROS was observed in MDMs infected with PRRSV at 48 h PI (Figure 7A).

## Discussion

PRRSV has a tropism for cells of the monocytic lineage expressing the surface molecule receptor CD163. Alveolar macrophages, as target cells and the primary replication site of PRRSV, have been one of the most widely used porcine cells for in vitro studies of PRRSV pathogenesis [36–38]. The available porcine blood monocytes do not express CD163, or express it at exceedingly low levels. The expression of CD163 increases with the cultivation of monocytes and this differentiation/aging enhances susceptibility to PRRSV infection [9]. Van Gucht et al. [39] showed that CD14-positive monocytes infiltrate the lungs of pigs after PRRSV infection. Monocytes in the lungs differentiate into CD163-positive macrophages [40]. In most studies, monocytes were cultivated in the presence of M-CSF/GM-CSF [11] or using LM-929 (murine fibroblast cells expressing murine M-CSF and GM-CSF) cell supernatant [9, 10, 41] to prepare MDMs. In the present study, MDMs were prepared without the addition of cytokines, similarly to Vicenova et al. [31] and García-Nicolás et al. [12]. The aim of the presented study was to compare the susceptibility of two different types of macrophages to the infection with PRRS virus and to co-infection with *H. parasuis.* Alveolar macrophages as resident cells provide one of the first lines of defence against microbes invading lung tissue. On the other hand, monocyte derived macrophages represent naive inflammatory cells accumulating at the site of inflammation [39, 40].

Sensitivity of macrophages to infection with PRRSV was the most significant observed difference between PAMs and MDMs. While alveolar macrophages were relatively resistant to cytopathogenic effect caused by PRRSV, monocyte derived macrophages were much more sensitive to PRRSV infection (Figures 2 and 3). A recent study by García-Nicolás et al. [12] showed decreased resistance to PRRSV infection of MDMs non-stimulated by cytokines. In contrast, IFN-γ or IFN-β pre-treated MDMs were protected against cytopathogenic effect caused by different strains of the PRRS virus (including strain Lelystad LVP23) [12].

Different responses of PAMs and MDMs to viral and bacterial infections were also reflected on the expression of pro-apoptotic (Bax, Bad and p53) and anti-apoptotic genes (Bcl-2) (Figure 5). Apoptosis is a strictly regulated mechanism of cell death and is highly modulated by both pro-apoptotic and anti-apoptotic cellular factors [16]. Bax is a pro-apoptotic protein normally occurring in the cytosol which, upon induction of apoptosis, translocates to mitochondria and induces cytochrome c release [42, 43]. In contrast, anti-apoptotic Bcl-2 is an integral membrane protein localized in mitochondria and has been shown to be capable of blocking spontaneous cytochrome c release [44, 45]. The function of another pro-apoptotic protein Bad is supposed to form a heterodimer with Bcl-2, inactivating it and thus allowing Bax triggered apoptosis [46]. The tumour suppressor p53 is activated by external and internal stress signals that promote its nuclear accumulation in an active form and stimulates a wide network of signals that act through two major apoptotic pathways [47, 48]. MDMs infected with PRRSV increased the expression of pro-apoptotic p53, Bad and Bax mRNA (Figures 5B–D). In contrast, expression rates of these genes and Bcl-2 were unchanged in PRRSV infected PAMs (Figures 5E–H). *H. parasuis* infected MDMs downregulated mRNA expression of pro-apoptotic p53 and Bad at 4 h PI (Figures 5B and C). *H. parasuis* as a gram negative bacterium has an outer membrane containing lipopolysaccharides (LPS) and LPS-mediated survival of macrophages has been previously observed by Lombardo et al. [49]. The expression of these genes may also be associated with a significantly decreased level of ROS production after infection with *H. parasuis* (Figure 7A) as was already described in PAMs [26]. Oxidative stress caused by ROS has been suggested as a mediator of the intrinsic pathway of apoptosis [13, 50]. On the other hand, increased mortality of MDMs may be related to an increased level of ROS production after infection with PRRSV (Figure 7A). These results are related to the data presented by Le and Kleiboeker [13] who demonstrated that oxidative stress caused by ROS production could play a central role in PRRSV-induced apoptosis of MARC-145 cells. In addition, higher expression of the surface molecule CD163 (Figure 1B), which serves as an essential receptor for PRRSV infection [4, 5], could affect the sensitivity of MDMs to PRRSV. Decrease production of ROS by *H. parasuis* and potential antioxidative mechanisms of bacteria was described previously in our study [26]. On the contrary, Fu et al. [51] demonstrated that *H. parasuis* induced ROS production in piglet mononuclear phagocytes after 3 and 6 h incubation with bacteria. This difference could be explained by different type of used cells (monocytes vs macrophages), different strain of *H. parasuis* used (strain SH0165 isolated from lung vs strain HP 132 strain isolated from brain) and different time of exposition to bacteria (0–2 h vs 3–6 h).

We had supposed that higher mortality of MDMs infected with PRRSV would result in decreased replication capabilities of PRRSV and reduced amounts of virus particles released into the culture supernatant compared to PRRSV-infected PAMs. Surprisingly, viral titres detected in MDMs culture supernatant and expressed as TCID_50_/mL were at similar levels as viral titres detected in the supernatant of PAMs (Table 2). In addition, PRRS virus released from MDMs remained stable over long periods of time, which could be due to the microenvironment created by decaying cells. Alternatively, surviving alveolar macrophages can effectively prevent virus replication and thereupon decrease viral load in the supernatant. Replication of virus was not affected by presence of *H. parasuis* in the case of simultaneously infected cells. On the other hand, the growth of *H. parasuis* was affected by higher mortality of MDMs after infection with PRRSV (Figure 4). Lower multiplication of *H. parasuis* in co-infected cells could be explained by absence of *H. parasuis* essential factor NAD which is produced by living cells [52].

MDMs infected with *H. parasuis* alone, but not in co-infection, formed multinucleated giant cells (Figure 6). These cells originate from fusion of macrophages and have been observed after infection by intracellular pathogens such as *Mycobacterium tuberculosis* [53] and *Burkholderia pseudomallei* [54]. MGCs, also called Langhans giant cells, are frequently present in granulomas with characteristic location of the nuclei at the cell periphery in an arcuate configuration [53]. Yanagishita et al. [55] investigated the possible roles of bacterial endotoxins on macrophage multi-nucleated giant cell formation in a mouse macrophage RAW 246.7 cell line. MGC formation could represent a unique cellular response, which macrophages engage when challenged with bacterial endotoxins [55, 56] or large foreign bodies that cannot be ingested [57]. MDMs when simultaneously infected with PRRSV and *H. parasuis* did not form multinucleated giant cells, which is partly associated with high mortality of MDMs after infection with PRRSV. Infection with PRRSV could therefore facilitate the development of a secondary bacterial infection by avoiding the formation of MGCs by macrophages.

In conclusion, concurrent infection of PAMs or MDMs with PRRSV and *H. parasuis* was performed in vitro and differences dependent on the macrophage type were described. Higher mortality of MDMs infected with PRRSV compared with infected PAMs was observed. MDMs infected with PRRSV also produced higher amounts of ROS and increased the expression of pro-apoptotic genes compared to PAMs. Higher sensitivity of MDMs to PRRSV infection, which is associated with limited MDMs survival and restriction of MGC formation, could contribute to the development of multifactorial respiratory disease of swine.

### Additional file

**Additional file 1.** **Comparison of CD163 expression on monocytes, MDMs and PAMs.** Expression of surface molecule CD163 (dark grey) was measured by flow cytometry. Appropriate control sample (light grey) for each analysed cell type is shown for autofluorescence demonstration. Data are presented as representative histograms.
