# Supplementary figures and images for: Concurrent infection with porcine reproductive and respiratory syndrome virus and Haemophilus parasuis in two types of porcine macrophages: apoptosis, production of ROS and formation of multinucleated giant cells
Source: Vet Res. 2017 May 4;48:28. doi: 10.1186/s13567-017-0433-6 (PMC5418695; doi:10.1186/s13567-017-0433-6)

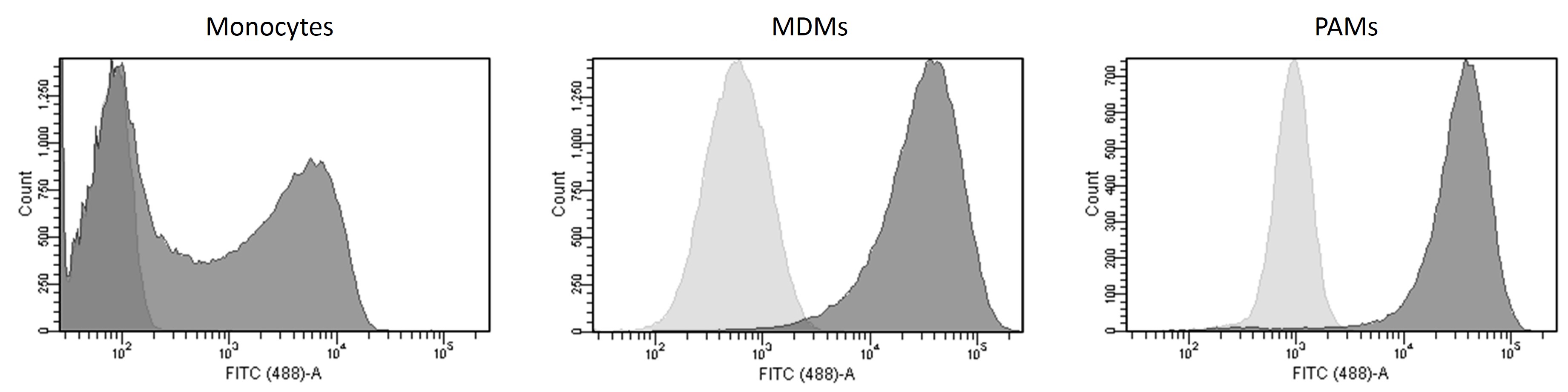

Supplement: Supplementary file 1 — Additional file 1. Comparison of CD163 expression on monocytes, MDMs and PAMs. Expression of surface molecule CD163 (dark grey) was measured by flow cytometry. Appropriate control sample (light grey) for each analysed cell type is shown for autofluorescence demonstration. Data are presented as representative histograms. [file 13567_2017_433_MOESM1_ESM.jpg]
